# Discarding Functional Residues from the Substitution Table Improves Predictions of Active Sites within Three-Dimensional Structures

**DOI:** 10.1371/journal.pcbi.1000179

**Published:** 2008-10-03

**Authors:** Sungsam Gong, Tom L. Blundell

**Affiliations:** Biocomputing Group, Department of Biochemistry, University of Cambridge, Cambridge, United Kingdom; Stanford University, United States of America

## Abstract

Substitutions of individual amino acids in proteins may be under very different evolutionary restraints depending on their structural and functional roles. The Environment Specific Substitution Table (ESST) describes the pattern of substitutions in terms of amino acid location within elements of secondary structure, solvent accessibility, and the existence of hydrogen bonds between side chains and neighbouring amino acid residues. Clearly amino acids that have very different local environments in their functional state compared to those in the protein analysed will give rise to inconsistencies in the calculation of amino acid substitution tables. Here, we describe how the calculation of ESSTs can be improved by discarding the functional residues from the calculation of substitution tables. Four categories of functions are examined in this study: protein–protein interactions, protein–nucleic acid interactions, protein–ligand interactions, and catalytic activity of enzymes. Their contributions to residue conservation are measured and investigated. We test our new ESSTs using the program CRESCENDO, designed to predict functional residues by exploiting knowledge of amino acid substitutions, and compare the benchmark results with proteins whose functions have been defined experimentally. The new methodology increases the Z-score by 98% at the active site residues and finds 16% more active sites compared with the old ESST. We also find that discarding amino acids responsible for protein–protein interactions helps in the prediction of those residues although they are not as conserved as the residues of active sites. Our methodology can make the substitution tables better reflect and describe the substitution patterns of amino acids that are under structural restraints only.

## Author Summary

Identification of residues responsible for a specific function of a protein can provide clues about the mechanism of action. Computational approaches to identifying functional residues have emerged as low-cost alternatives to experimental methods by providing fast and large-scale analyses. Moreover, the demand for such approaches is increasing as more sequences become available from genome sequencing projects. Here, we focus on the use of CRESCENDO to identify functional residues in proteins of known structure by comparing the amino acid substitutions observed in a family of proteins with those predicted on the basis of the protein structure. CRESCENDO uses Environment Specific Substitution Tables, or ESSTs, which define the way that accepted amino acid substitutions are influenced by the local structural environment. We describe how the calculation of ESSTs can be improved by using only amino acids that are not involved in catalytic activity, metal or ligand binding, nucleic acid or protein interactions, and other molecular functions. Our new substitution table can better describe the degree of amino acids substitutions that are under structural restraints. It should be of value in all applications of ESSTs, including their use in sequence–structure homology recognition, structure validation, and structure prediction in addition to their use in the identification of functional residues. These approaches should enhance the understanding of protein structure and function, which is critically important in the postgenomic era.

## Introduction

Proteins existing in living organisms have been selected through the process of evolution. However, much of the amino acid variation between orthologues appears to be selectively neutral [1] as far as the whole organism is concerned and accepted amino acid substitutions result in equal fitness. It has been long understood that the rate and nature of accepted mutation or substitution is different for the 20 amino acids in a protein [2]–[5]. Indeed the different substitution rates and patterns for the 20 amino acids were first quantified by Margaret Dayhoff as the PAM (Percentile Accepted Mutation) matrix in 1970s [2], which measures the point mutation for every 100 amino acids. The methodology was further developed by Henikoff *et al.* [3] to reflect more divergent relationships of protein sequences. The BLOSUM62 is now recognized as a standard measure of substitution rate for the 20 amino acids in the sequence comparisons. Jones *et al.* [4] introduced a fast and automated approach based on a maximum parsimony counting method and Whelan *et al.* [5] applied a maximum-likelihood method to estimate the rate for amino acid replacement. All these substitution models are based on the sequence alignments of closely related protein families.

Orthologous protein families (or superfamilies) are assumed to be diverged from a common ancestor by accepting mutations that are selectively neutral. The rate of evolution [1] is assumed to be constant over evolutionary time [6],[7] and so evolutionary distances can be measured by analysing the substitutions of amino acids. The degree of conservation and the nature of substitutions of amino acids will be under many evolutionary restraints. One of those is dependent on the need to retain the protein tertiary structure and usually expressed as a tendency to maintain the local structural environments of individual amino acids [8].

The Environment Specific Substitution Table (ESST) is a substitution table that considers structural restraints in the calculation of substitution patterns. Overington *et al.* [9],[10] first calculated ESSTs from a set of homologous protein families whose three-dimensional structures were available. The rationale behind ESSTs is that the acceptance of substitution of an amino acid in an orthologous family is subject to its local tertiary environment. The local structural environments of amino acids include (1) main-chain conformation and secondary structure, (2) solvent accessibility, and (3) hydrogen bonding between side-chain and main-chain. 64 ESSTs can be derived from a combination of structural features; four from secondary structures (α-helix, β-strand, coil and residue with positive ϕ main-chain torsion angle), two from solvent accessibility (accessible and inaccessible), and eight (2^3^) from hydrogen bonds to main-chain carbonyl or amide or to another side-chain. These combinations of structural features restrict possible substitutions of an amino acid and give rise to distinct patterns of substitution.

The ESST was improved and updated by Shi *et al.* [11] in 2001 by the use of the following features: (1) a clustering scheme to correct sampling bias, (2) a smoothing procedure to correct data sparsity, (3) using only high resolution structures in the alignments as a source of substitution matrices and (4) reduction of the bias caused by non-structural restraints. The last feature was designed to separate functional restraints from structural restraints when generating ESSTs. Because ESSTs take into account only structural environments, substitutions where the amino acids are conserved for functional reasons should not be counted in the calculation of matrices. Shi *et al.* took two kinds of functional residues into account to eliminate non-structural restraints which may cause a bias in the ESST. They were (1) residues involved in domain-domain interactions and (2) those interacting with ligand. Such residues were masked in the alignment files and were not taken into account in the substitution counts. However, the masking appeared to have very little impact on the performance of FUGUE [11]. Chelliah *et al*. [12] further developed ESSTs by introducing functional restraints, particularly in enzymes, on amino acid substitutions as a new environment in addition to 64 structural environments. They measured the Euclidean distance between every amino acid and the known functional residues and compared the degree of conservation in terms of the proximity with the functional residues. Their ESST, known as the function-dependent ESST, showed improvements in sequence to structure homology recognition.

Compared with traditional substitution tables (PAM, BLOSUM) derived from sequence information only, ESSTs were shown to give more precise and discriminating measures of substitution probabilities [13]. ESSTs have been shown to be useful in applications to secondary structure prediction [13] and sequence-structure homology recognition [14],[15]. Recently, CRESCENDO [8] has been successful in prediction of functional residues by comparing the observed substitution patterns for amino acids which are under both functional and structural constrains with those that are predicted on the basis of structure alone.

Here we investigate the impacts of various functional restraints on the conservation of amino acids in three-dimensional structures. The functional residues are divided into four categories. They are residues involved in (1) protein–protein interaction, (2) protein–nucleic acid interaction, (3) protein–ligand interaction, and (4) catalytic reaction at enzyme active sites. Such residues will be under greater pressure to be conserved throughout the evolution process where they remain critically important to the activity of protein and thus the selective advantage of the organism. We measure the degree of functional residue conservation by masking the locations in the alignment file and then discarding them in the calculation of substitution probabilities. The substitution models are compared with the non-masking model which counts those functional residues in the calculation of substitution probabilities. We measure relative contributions of four categories of functional residues by making several masking tables in combinatorial fashion. We test our substitution models by performing computational experiments using CRESCENDO [8] which is a program predicting functional residues from known three-dimensional structures of proteins and which should be more sensitive to the accuracy of the predicted substitution tables than FUGUE [11]. We show that our new ESST can find 16% more functional residues compared with the ESST of Shi *et al.* [11] for the same test-set. The new ESST is different from previous ones in that we cover a broader range of protein families, we take into account more three-dimensional structures and we consider a wider variety of functional residues which may bias amino acid substitution patterns.

## Results/Discussion

### Locating Functional Residues in Three-Dimensional Structures

Four categories of functional residues are considered in this study (Table 1). The first category of functional residues comprises catalytic residues of enzyme active sites, which are strongly conserved in orthologous families and often across superfamilies. CSA [16] and “ACT_SITE” records in UniProt [17] were used. The Catalytic Site Atlas (CSA) is a database of enzyme active sites and catalytic residues of enzymes whose 3D structures are available. It provides two types of entries: (1) original hand-annotated entries derived from the primary literature and (2) entries homologous to one of the original entries by sequence similarity. We took into account only the hand curated entries for reasons of reliability. The second category comprised amino acids involved in protein–protein interactions. Data concerning protein interactions were retrieved from InterPare [18] which is a database for interacting interfaces between protein domains. InterPare uses SCOP [19] as a domain definition and detects interacting domain pairs if there are at least five pairs of residues which fall within 5 Å distance between two adjacent domains. Residues interacting with nucleic acids comprise the third category. BIPA (S. Lee, unpublished) and “DNA_BIND” records in UniProt were used for this category. BIPA is a database for protein–nucleic acid interactions, which defines the atomic interactions using a distance threshold of 5 Å for van der Waals contacts, and HBPLUS [20] default options for hydrogen bonds and water mediated hydrogen bonds. The final category comprises the ligand-binding residues. For this information, the following UniProt feature annotations were used: “BINDING”, “METAL”, “NP_BIND”, and “CA_BIND” (see Table 1 for details).

**Table 1:** Four Categories of Functional Residues Considered in this Study.

Functional Category	Database	Feature Identifier	Description	Masking Type				URL
				A	B	C	D	
**Protein–protein Interaction**	**InterPare**	N/A	Database of domain–domain interaction interface	√		√		http://interpare.net
**Catalytic activity**	**CSA**	N/A	Database documenting enzyme active sites and catalytic residues in enzymes of 3D structure	√	√		√	http://www.ebi.ac.uk/thornton-srv/databases/CSA/
	**UNIPROT**	**ACT_SITE**	Amino acid(s) involved in the activity of an enzyme	√	√		√	http://www.uniprot.org
**Protein–nucleic acid interaction**	**BIPA**	N/A	Database of protein–nucleic acid interactions	√	√	√		N/A
	**UNIPROT**	**DNA_BIND**	Extent of a DNA-binding region	√	√	√		http://www.uniprot.org
**Protein–ligand interaction**	**UNIPROT**	**BINDING**	Binding site for any chemical group (co-enzyme, prosthetic group, etc.)	√	√	√		http://www.uniprot.org
		**CA_BIND**	Extent of a calcium-binding region	√	√	√		
		**NP_BIND**	Extent of a nucleotide phosphate-binding region	√	√	√		
		**METAL**	Binding site for a metal ion	√	√	√		

The versions of CSA [16] and UniProt [17] were 2.2.7 and 12.2, respectively. InterPare [18] was based on SCOP [19] version 1.71. The “Feature Identifier” is only for UniProt annotations. (A: all masking, B: no protein–protein interaction, C: no active sites, D: active-site only.)

The data from InterPare, CSA and BIPA are based on three-dimensional structures of proteins. Hence, those functional residues can be easily identified and mapped into PDB entries using chain and residue numbers as unique identifiers. However, as the functional feature annotations from UniProt are based on sequence information, they are required to be mapped into their corresponding PDB entries. For this purpose, we developed a mapping protocol named “double-map” to align a sequence from UniProt with that of PDB at the residue level. This mapping protocol is critically important as we should find and mask the exact functional residues from the structural alignment. The detailed algorithm of double-map is described in Material and Methods.

### Structure Alignments and New Environment Specific Substitution Table

The new Environment Specific Substitution Table (ESST) was built based on the alignments of three-dimensional structures of proteins which belong to the same protein family. We used PDB as a source for the three-dimensional structures of proteins and SCOP as the definition of protein families and domains. SCOP version 1.71, which was used in this study, classifies 3004 families and 75930 domains from 27599 PDB entries. For each SCOP family, domains were clustered with sequence identity of 80% or more, after pre-processing the structure data (see Materials and Methods for details). Within a cluster defined in this way, a structure having the best resolution was selected as a representative for the structure alignments. This process yielded 1187 SCOP families having 5833 domains from 4309 PDB entries. These final alignments, which are shown as “ALL” in the matrix type of Table 2, were used as a source for the calculation of substitution tables.

**Table 2:** 17 ESSTs and the Number of Functional Residues Masked from the Alignments.

Alignment Source	Number			Matrix Type	Masking Type	Masking Residues^b^	%Mask^c^
	Family	Structure	Residue^a^				
**HOMSTRAD**	177	706	146,437	**OLD**	X	0	0.00
					J	2,048	1.40
					B	4,601	3.14
					R	4,601	3.14
**SCOP**	221	902	235,588	**ENZ**	X	0	0.00
					A	37,808	16.05
					B	6,195	2.63
					C	36,265	15.39
					D	1,615	0.69
					R	37,808	16.05
	566	2,556	384,618	**NOENZ**	X	0	0.00
	1,187	5,833	1,096,027	**ALL**	X	0	0.00
					A	198,411	18.10
					B	21,830	1.99
					C	191,377	17.46
					D	1,840	0.17
					R	198,411	18.10

New ESSTs were based on the structure alignments of SCOP families [19]. ENZ is 221 enzyme-specific SCOP families which contain at least one ACT_SITE annotation of UniProt [17] or hand-curated CSA entry [16]. NOENZ is the opposite of ENZ. NOENZ does not even contain the predicted entries of CSA. ALL is the final alignment source obtained from the filtering process (see Materials and Methods). The masking sources of A, B, C, and D are in Table 1. X is for non-masking and R is for random-masking. R is set as a control to see the significance of removing functional residues from the substitution models. The ESST of Shi *et al.* (OLD-J) [11] is based on 177 HOMSTRAD families which consist of 706 structures. It masks 2,048 resides which are involved in (1) interaction with heteroatoms and (2) domain–domain interaction. OLD-X and OLD-R is non-masking and random-masking model of J.

a Number of all residues.

b Number of masking residues.

c %Mask = number of masking residues/number of all residues*100.

Table 2 shows 17 ESSTs and compares the numbers of structures and the functional residues masked from the alignments. There are four matrix types which differ in the alignment source; OLD, ENZ, NOENZ and ALL. “OLD” is based on the 177 HOMSTRAD families, from which the ESST of Shi *et al.* [11] was derived. “ENZ” is for the 221 enzyme-specific SCOP families whose members contain at least one “ACT_SITE” residue or CSA hand-curated entry. “NOENZ”, the opposite of “ENZ”, does not contain any “ACT_SITE” annotations or CSA entries at all. These two matrix types are prepared in order to assess the effect of alignment sources in the substitution patterns of amino acids. “ALL” is based on 1187 SCOP families described above. SCOP families that belong to ENZ and NOENZ are subsets of ALL type and do not overlap as they include different SCOP families. Each matrix type is further divided into several subtypes (A, B, C, and D) which differ in the masking sources of functional residues (see Table 1). This is to investigate the effect of a specific category of functional residues by comparing the differences in the substitution patterns. For example, the effect of masking enzyme active sites can be measured by calculating the difference between two matrices D and X, because X does not mask any functional residues whereas D masks only active site residues. We made random-masking models (R), in order to assess the value of masking models in benchmarking the new ESSTs. Our new ESSTs mask more functional residues than the ESST (J) of Shi *et al.*, because our models take into account a broad range of structural families and functional residues. ESSTs and structure alignments in Table 2 are available from http://www-cryst.bioc.cam.ac.uk/ESST.

### Differences between Substitution Tables: The Effects of Alignment Source and Masking

**Figure 1:**
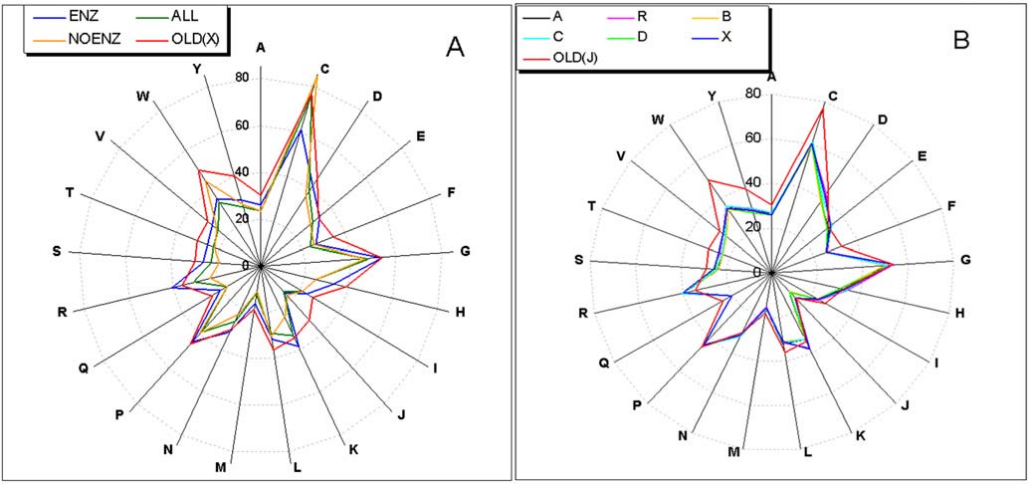
Probabilities of Residue Conservation for 21 Amino Acids. The probability of residue conservation (P_CONS_) was averaged for the diagonal axis of substitution tables. (A) P_CONS_ of three matrix-types (ENZ, NOENZ and ALL) are compared with the OLD. Non-masking models (X) were used for three matrix-types and OLD to see the effect of alignment source. (ENZ: enzyme-specific 221 SCOP families, NONENZ: non-enzymes, ALL: all the alignments, OLD: non-masking ESST of Shi *et al.* [11]. See Table 2 for details.) (B) Five masking tables and one non-masking table are compared with the ESST of Shi *et al.* [11]. Masking and non-masking tables are from the 221 enzyme-specific alignments (ENZ). Masking sources of A, B, C, and D are listed in Table 1. (R: random-masking, X: non-masking.)

Our new ESSTs differ from those of Shi et al. [11] in the source of structure alignments and the categories (and the number) of functional residues removed from the alignments. The differences between 17 substitution tables were measured and investigated in terms of 1) the conservation probability of amino acids (P_CONS_) and 2) the distance (DIST) between ESSTs (see Materials and Methods). We first looked at the different sources of structure alignments to assess their effects on the amino acid conservation in the substitution table. For this purpose, the non-masking models (X) from four alignment sources (OLD, ENZ, NOENZ and ALL) were compared. Figure 1A plots the P_CONS_ of 21 amino acids (P_CONS_ in Table S1). The conservation probability in the figure is averaged over the diagonal entries (i.e. those amino acids which are not substituted) from 64 ESSTs for each model. The overall degree of conservation is 28.93, 29.10, 32.08, and 36.73% for NOENZ, ALL, ENZ and OLD respectively (see Table S1 for details). All the amino acids in OLD-type are more conserved than those of ALL-type. We are aware that the number of structures and families in the alignment may affect the P_CONS_. In addition, the definition of protein families and domains of HOMSTRAD is more stringent than those of SCOP. This will make the sequences less divergent and the alignments more conserved. The distance of substitution tables (Table S2) shows that NOENZ and ENZ are the most distant (507) among four tables and NOENZ and ALL are the closest. This is clear as NOENZ and ENZ do not share nay families but all the families in NOENZ belong to ALL. Figure 1A shows that amino acids R, K, H and S of ENZ-type are more conserved than those from NOENZ by 17, 14.2, 8.5 and 7%, respectively. However, C and W from ENZ are less conserved than those of NOENZ by 24% and 9%.

Figure 1B shows P_CONS_ of amino acids from the same source of alignment (ENZ) but having different masking types (A, B, C and D), being compared with non-masking (X), random-masking (R) and ESST of Shi *et al.* (OLD-J). Overall, the differences of P_CONS_ among the tables are less clear than the differences shown in Figure 1A. In addition, Table S2 shows that the distances (DIST) between tables of different masking types, but having the same alignment source, are smaller than the distances of tables from the different alignment sources. This explains why the variations of P_CONS_ and DIST between tables are more affected by the source of alignments than the masking sources. However, the relationship between P_CONS_ (or DIST) and the number of masking residues (%Mask) could be clearly understood by the Spearman's rank correlation between two (see Table 3). The more we mask functional residues (%Mask) from the alignments, the smaller P_CONS_ gets and the greater the difference as measured by DIST between the substitution tables. We found that the correlation between P_CONS_ and %Mask (−0.3) was not made more distinctive by removing residues involved in protein–protein interactions. A-type masks 13.4% and 16.9% many more residues than B-type in ENZ and ALL, respectively, where the discrepancies lie in the protein–protein interactions as B does not include InterPare as masking sources. However, the average P_CONS_ of A is bigger than B, although A masks much more residues than B. This becomes much clearer on looking at the P_CONS_ of A and D where the difference is in residues annotated as CSA and ACT_SITE. The P_CONS_ of D is bigger than A, although D masks many fewer residues than A. The result shows that the residues involved in protein–protein (or domain-domain) interactions are not as conserved as residues responsible for the catalytic activity of enzymes. From P_CONS_ of ENZ-D and ENZ-X (Table S1), which differ in active sites as masking source, we observe that active site residues J, D, H and E are most conserved throughout enzyme families, where H is the most abundant amino acid annotated as ACT_SITE or CSA followed by D, E, and J.

**Table 3:** Rank Correlation.

	P_CONS_	Z-Score	SENS	DIST	%Mask
**P_CONS_**	1	−0.85	−0.93	−0.38	−0.30
**Z-score**		1	0.95	0.54	0.45
**SENS**			1	0.48	0.45
**DIST**				1	0.29
**%Mask**					1

Spearman's rank correlations were calculated between the variables of P_CONS_, Z-score, SENS, DIST, and %Mask. See Materials and Methods for the definition of Spearman's rank correlation. %Mask is from Table 2. Z-Score and SENS are from Table 5. DIST is from the first row of Table S2. P_CONS_ is from the bottom line of Table S1. P_cons_: probability of residue conservation, Z-score: average Z-score 602 active sites, SENS: sensitivity, DIST: distance between two ESSTs, %Mask: percentage of discarded functional residues.

### Benchmarking Design

The performance of the new ESSTs was benchmarked by using CRESCENDO [8], which is a program for predicting functional residues given a three-dimensional structure. The rationale behind CRESCENDO is to distinguish functional restraints from structural restraints, both of which give rise to the conservation of amino acids in the evolutionary process. For example, amino acids in the core region of a protein are conserved or conservatively varied in order to maintain an appropriate structure (and ultimately function) whereas the catalytic triad of a protease, such as CYS-HIS-ASP, is conserved to maintain the functional properties of the enzyme family. CRESCENDO quantifies the degree of amino acid conservation by measuring (1) the observed value based on the alignment to which a queried protein sequence belongs and (2) the expected value calculated by using ESST. Note that the first value reflects both structural and functional restraints, whereas the latter only reflects the structural restraints because ESST, by definition, only takes structural environments into account. The overall difference between the two is converted into Z-score (or CRESCENDO score) which can represent extra restraints—probably functional—on the process of evolution. Hence, the more accurate the ESST, the less good the agreement between the probabilities of conservation observed and that predicted on the basis of the structure of the protein alone. CRESCENDO can be a good benchmarking tool for the evaluation of new ESSTs, because more functional residues are masked than the old ESST. In addition, we can identify relative contributions of four masking resources on the performance of ESSTs. The benchmarking was designed to investigate the following two questions. (1) How well can a new ESST identify functional residues compared with the ESST of Shi *et al.* which is used currently as the default by CRESCENDO? (2) If there is any improvement, what makes the improvement?

From 221 enzyme-specific SCOP families for ENZ in Table 2, one third (73 SCOP families) was selected as a test-set and the rest were used to make benchmarking-ESSTs for ENZ. The test-set consists of 339 SCOP domains having 81,410 residues in total. Out of 81,410 residues, 602 residues are active sites (ACT_SITE or CSA), 11,917 residues are annotated by InterPare, 194 residues for nucleic-acid interactions and 1,348 residues are involved with ligand interactions. They are the true functional residues that we are trying to predict using CRESCENDO in order to evaluate the performance of our new ESST. In our analysis we took only the first cluster as the predicted residues. The performance of our new ESST was compared with that of the old in terms of detecting functional residues. Note that, for both ENZ and ALL types, the 73 SCOP families in the test-set were removed from the original ESST. The benchmarking ESSTs were renamed as At, Bt, Ct, Dt, Rt, and Xt to distinguish them from the original new ESSTs which are A, B, C, D, R, and X, respectively. This is in order to make our benchmarking an unbiased blind test by removing sequences in the test-set which might affect the benchmarking results. In the case of OLD and NOENZ, the original masking types were used in the benchmarking process as they do not contain SCOP families in the test-sets. The test-sets and benchmark results are accessible from http://www-cryst.bioc.cam.ac.uk/ESST.

### Performance of New ESSTs in Detecting Functional Residues

Table 4 shows the average Z-score of CRESCENDO for 602 active sites, 11,917 PPI residues, 194 residues for protein–nucleic acid interactions (PNI) and 1348 residues responsible for interaction with ligands (PLI) along with the P-values for the predicted residues. The P-value demonstrates that the Z-score of the predicted residues is different from the randomly selected residues with a 0.09 level of significance. In other words, we can say that the predicted residues of CRESCENDO are far from the random within 0.09 error rate. The Z-scores for all the residues (81,410) in the test-sets are compared with those of functional residues predicted by CRESCENDO. The average Z-score of all the residues is near zero, regardless of masking types, which means there are no differences between the probabilities of residue conservations observed in the alignments and those predicted by ESST. However, the Z-scores for 602 active sites range between 0.48 and 0.93 depending on the matrix types and the masking sources. This observation suggests there are extra restraints which make the active sites more conserved in families of homologous proteins. The Z-scores of 1,348 PLI (Protein–Ligand Interaction, see Table 4) residues also imply that they are under extra restraints other than structural reasons. On the other hand, the average Z-scores for PPI and PNI residues are much smaller than that of 602 active sites. This may suggest that residues at protein–protein interfaces are under less strong restraints than residues responsible for the catalytic activity. However, there is strong evidence that sub-regions in protein interfaces—so called hot spots—are energetically more important and may be under stronger restraints in evolution [21],[22].

**Table 4:** Z-Score of CRESCENDO for Functional Residues.

Matrix Type	Masking Type	Average Z-Score						Ratio^g^	P-Value^h^
		All^a^	Predicted^b^	Active Site^c^	PPI^d^	PNI^e^	PLI^f^		
**OLD**	X	0.00063	1.396	0.480	0.0250	0.055	0.449	0.78	0.081
	R	0.00067	1.402	0.483	0.0249	0.052	0.450	0.79	0.080
	J	0.00062	1.410	0.612	0.0284	0.055	0.461	1.00	0.079
	B	0.00065	1.420	0.734	0.0274	0.059	0.490	1.20	0.078
**ENZ**	Xt	0.00060	1.387	0.635	0.0042	0.024	0.426	1.04	0.083
	Rt	0.00060	1.387	0.652	0.0067	0.025	0.431	1.06	0.083
	Ct	0.00063	1.413	0.734	0.0100	0.025	0.427	1.20	0.079
	Dt	0.00062	1.399	0.772	0.0078	0.051	0.428	1.26	0.081
	At	0.00063	1.423	0.858	0.0143	0.056	0.433	1.40	0.077
	Bt	0.00064	1.411	0.870	0.0086	0.068	0.447	1.42	0.079
**NOENZ**	X	0.00063	1.420	0.835	0.0046	0.099	0.508	1.36	0.078
**ALL**	Xt	0.00063	1.414	0.696	0.0085	0.068	0.489	1.14	0.079
	Rt	0.00064	1.415	0.771	0.0065	0.075	0.501	1.26	0.079
	Dt	0.00066	1.412	0.798	0.0055	0.078	0.495	1.30	0.079
	At	0.00064	1.433	0.860	0.0159	0.069	0.495	1.41	0.076
	Ct	0.00067	1.436	0.893	0.0155	0.077	0.515	1.46	0.076
	Bt	0.00068	1.435	0.936	0.0073	0.086	0.518	1.53	0.076

The average Z-scores are shown for four categories of functional residues in the test-sets: catalytic activity, protein–protein interactions, protein–nucleic acid interactions, and protein–ligand interactions. The test-sets consist of 73 SCOP families, which is one third of SCOP families in ENZ (see Table 2).

a Total number of residue from test-sets (81,410).

b Residue predicted by CRESCENDO.

c Active-site residues (602).

d Protein–protein interaction sites (11,917).

e Protein–nucleic acid interaction sites (194).

f Protein–ligand interaction sites (1,348).

g Ratio of Z-score at the active site residues compared with that of OLD-J.

h P-value (right-tail) of the predicted residues.

**Figure 2:**
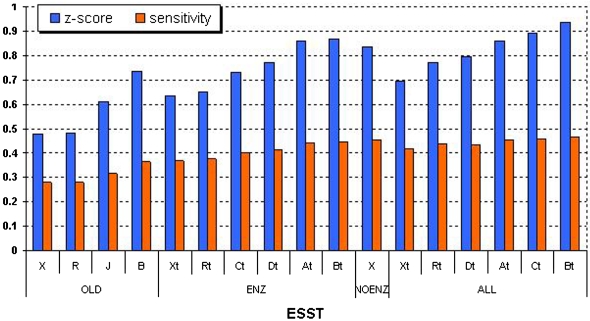
Performance of 17 ESSTs on Detecting Active Site Residues. Z-score (blue) and sensitivity (red) are plotted against 17 ESSTs. Z-score is averaged for 602 active-site residues in the test-sets (see text). Z-score and sensitivity (SENS) are highly correlated (0.95 in Spearman's rank correlation, Table 3). If any SCOP families in the test-sets are included in 17 ESSTs, they are removed from the ESSTs to avoid any bias. Those benchmarking ESSTs are marked by ‘t’ (e.g., At, Bt, Ct and Dt) to distinguish from the original. Z-score and SENS of non-masking (X) and random-masking (R) tables are always lower than those of masking models (At, Bt, Ct, and Dt) within the same matrix type (OLD, ENZ, ALL). All the masking-tables outperform the ESST of Shi *et al.* (J) [11].

**Figure 3:**
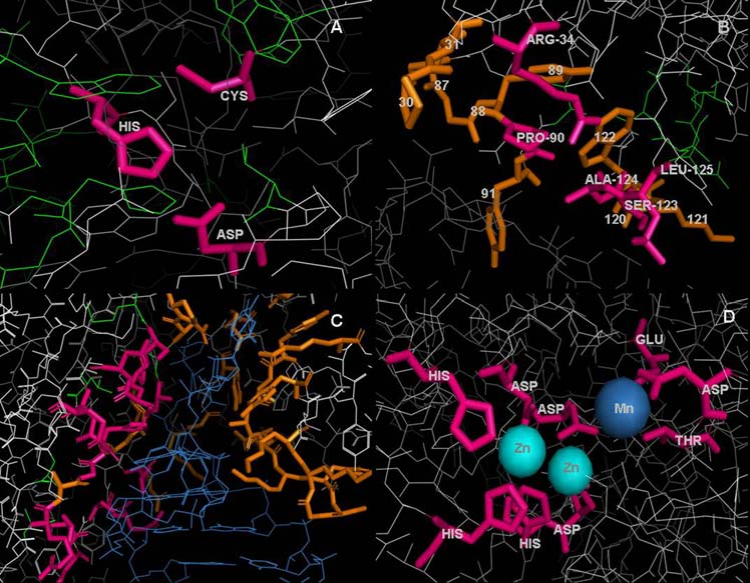
Predicting Four Categories of Functional Residues by CRESCENDO. Four case-studies of predicting functional residues are shown; (A) active-sites, (B) PPI (protein–protein interaction), (C) PNI (protein–nucleic acid interaction, (D) PLI (protein–ligand interaction). SCOP domains d1evua4 [23], d1i7kb_ [24], d1k8wa5 [33] and d1ed9a_ [34] were used for A, B, C, and D, respectively. True positives (TP) are coloured in pink, false negatives (FN, missing residues) in orange and false positives (FP) in green. TP and FN are shown as sticks (bold-frame). (A) Cysteine protease. CRESCENDO predicted 27 residues as functional residues. All three (CYS-314, HIS-373 and ASP-396) catalytic residues were correctly identified. ALL-B type ESST (see Table 2) was used in this figure. FP (green) are clustered around the three real active sites (pink). (B) Ubiquitin conjugating (UBC) enzyme. 12 residues were predicted by CRESCENDO using ALL-A ESST. Five (coloured in pink) were correctly identified among 14 residues annotated as PPI residues. Interacting partner (A chain of 1i7k) is placed at the bottom and coloured in gray. The solvent accessible surface areas (SASA) for five TP are as follow; ARG-34 (35.64), PRO-90 (4.12), SER-123 (4.74), ALA-124 (0.55), LEU-125 (72.39). SASA for 9 FN are as follow; PRO-30 (77.26), VAL-31 (24.02), SER-87 (110.40), GLY-88 (16.05), TYR-89 (0.01), TYR-91 (58.29), GLU-120 (108.68), LYS-121 (113.96), TRP-122 (7.20). The SASA is from InterPare [18]. (C) Pseudouridine synthase. BIPA (S. Lee, unpublished) annotates 43 residues as PNI. 14 residues were TP (coloured in pink) among 20 residues predicted by CRESCENDO. ALL-D was used as ESST. DNA is coloured in blue. (D) Alkaline phosphatase. UniProt annotates 9 residues as metal-binding (METAL), which were all correctly identified by CRESCENDO among 30 predicted residues. ALL-B was used as ESST. ZN (zinc) and MG (magnesium) are coloured in cyan and blue, respectively.

In Table 5, the performance of 17 ESSTs is compared in terms of recognizing 602 active-site residues. SENS, SPEC and COV were measured using the ratios of TP (true positive), FP (false positive), FN (false negative) and TN (true negative) (see Material and Methods for the definitions). The Z-score and SENS are plotted together in Figure 2; they are highly correlated having 0.95 Spearman's rank correlation score (Table 3). As shown in Figure 2, the average Z-scores and SENS of non-masking (X) and random-masking (R) models are always less than those from masking-models (A, B, C, and D) within the same matrix type. This clearly shows that the position of masking is significant and discarding the substitution counts of functional residues from the substitution table can increase the performance of CRESCENDO by making ESST less dependent on the substitution patterns of the residues under functional restraints. This result is clearer from the rank correlation (0.45) between %Mask and SENS in Table 3. In addition, our new masking models (A, B, C and D) outperform the ESST of Shi *et al.* (J) and even the non-masking model (ENZ-X, NOENZ-X and ALL-X) outperform J (see Figure 2 and Table 5). This can be explained in terms of P_CONS_ and SENS; the average P_CONS_ is highest in the order of J, followed by ENZ-X, ALL-X and NOENZ-X, but the performance (SENS) is exactly the reverse order of P_CONS_. Figure 3A shows an example of predicting active sites of a SCOP domain d1evua4 (a domain in the A chain of PDB 1evu, [23]) which is a cysteine proteinase containing three active site residues annotated by UniProt. Three active site residues (CYS-314, HIS-373 and ASP-396) could be identified only by ALL-type ESSTs (ALL-B and ALL-C) which are highly ranked in Figure 2. This is probably because P_CONS_ of ALL is lower than that of ENZ and OLD for the local environments of the three catalytic residues.

**Table 5:** Performance of 17 ESSTs on Detecting Active Sites.

Matrix Type	Masking Type	TP	FP	FN	TN	SENS	SPEC	COV	F-Measure
**OLD**	X	168	4832	432	75976	0.28	0.9401	0.0336	0.060
	R	168	4830	432	75978	0.28	0.9401	0.0336	0.060
	J	189	4877	411	75931	0.315	0.9395	0.0373	0.067
	B	219	4888	381	75920	0.365	0.9394	0.0429	0.077
**ENZ**	Xt	221	4942	379	75866	0.3683	0.9387	0.0428	0.077
	Rt	225	4968	375	75840	0.375	0.9384	0.0433	0.078
	Ct	240	4870	360	75938	0.4	0.9396	0.047	0.084
	Dt	248	4977	352	75831	0.4133	0.9383	0.0475	0.085
	At	264	4805	336	76003	0.44	0.9404	0.0521	0.093
	Bt	270	4984	330	75824	0.45	0.9382	0.0514	0.092
**NOENZ**	X	273	5234	327	75574	0.455	0.9351	0.0496	0.089
**ALL**	Xt	249	5283	351	75525	0.415	0.9345	0.045	0.081
	Dt	259	5285	341	75523	0.4317	0.9345	0.0467	0.084
	Rt	262	5246	338	75562	0.4367	0.935	0.0476	0.086
	At	273	5150	327	75658	0.455	0.9362	0.0503	0.091
	Ct	277	5136	323	75672	0.4617	0.9363	0.0512	0.092
	Bt	282	5187	318	75621	0.47	0.9357	0.0516	0.093

Out of 81,410 residues in the test-sets, 602 residues are annotated as “ACT_SITE” by UniProt [17] or CSA [16]. For those active sites, CRESCENDO [8] could either correctly predict (TP) or fail to predict (FN) (see text). Two active sites of ‘d7odca1’ (A chain of PDB 7odc), which is a SCOP domain in the test-sets, was discarded as of an internal error; hence, 600 active sites either in the TP or FN. The number of predicted residues is same as the sum of TP and FP for each ESST type. Note that residues only from the first cluster of predicted residues (rank 1) were considered in this analysis. TP: True Positive, FP: False Positive, FN: False Negative, TN: True Negative, SENS: Sensitivity, SPEC: Specificity, COV: Coverage.

Table 6 shows the recognition performance for 11,917 PPI residues with the same measurements (TP, FP, FN, and TN) in Table 5. Four masking substitution tables of ALL-matrix could detect more PPI residues than that of Shi *et al.* (J), but not all tables in ENZ-matrix outperform J. Regardless of matrix types and masking types, the sensitivity (SENS) of detecting PPI residues is much lower than those for detecting active site residues. We think that this arises from the average Z-score for PPI residues (see Table 4) which is close to zero, suggesting less strong evidence for extra restraints. Figure 3B shows an example of predicting PPI residues of a SCOP domain d1i7kb_ (B chain of PDB 1i7k, [24]) which is a ubiquitin conjugating (UBC) enzyme containing 14 residues interfacing with the A chain. Using ALL-A, CRESCENDO predicted 12 residues of which five were correct PPI residues (true positive, coloured in pink in Figure 3B). Among the nine missing residues (orange), PRO-30, SER-87, TYR-91, GLU-120 and LYS-121 were highly accessible (more than 50 Å^2^) to solvent in the complex whereas five true positives had relatively small solvent accessible area (see Figure 3B for details). Thus, as expected, residues within the protein–protein interaction interface which are partially accessible are less conserved and more difficult to identify by CRESCENDO. Table S3 contains benchmark results for detecting residues interacting with nucleic acids and ligands. The sensitivity is better than the benchmarking results of recognizing PPI residues but still less than that of detecting active site residues. Figure 3C and 3D show examples of predicting residues interacting with nucleic-acids and ligands, respectively (see Figure 3 for details).

**Table 6:** Performance of ESSTs on Protein–Protein Interaction Residues.

Matrix Type	Masking Type	TP	FP	FN	TN	SENS	SPEC	COV	F-Measure
**OLD**	B	931	4176	10986	65317	0.0781	0.8560	0.1823	0.1094
	R	934	4064	10983	65429	0.0784	0.8563	0.1869	0.1104
	X	939	4061	10978	65432	0.0788	0.8563	0.1878	0.1110
	J	939	4127	10978	65366	0.0788	0.8562	0.1854	0.1106
**ENZ**	At	906	4163	11011	65330	0.0760	0.8558	0.1787	0.1067
	Ct	908	4202	11009	65291	0.0762	0.8557	0.1777	0.1067
	Xt	921	4242	10996	65251	0.0773	0.8558	0.1784	0.1078
	Rt	925	4268	10992	65225	0.0776	0.8558	0.1781	0.1081
	Dt	960	4265	10957	65228	0.0806	0.8562	0.1837	0.1120
	Bt	973	4281	10944	65212	0.0816	0.8563	0.1852	0.1133
**NOENZ**	X	893	4614	11024	64879	0.0749	0.8548	0.1622	0.1025
**ALL**	Xt	930	4602	10987	64891	0.0780	0.8552	0.1681	0.1066
	Bt	953	4516	10964	64977	0.0800	0.8556	0.1743	0.1096
	Dt	963	4581	10954	64912	0.0808	0.8556	0.1737	0.1103
	Rt	980	4528	10937	64965	0.0822	0.8559	0.1779	0.1125
	Ct	1000	4245	10917	65248	0.0839	0.8567	0.1907	0.1165
	At	1003	4420	10914	65073	0.0842	0.8564	0.1850	0.1157

11,917 residues are annotated by InterPare [18] out of 81,410 residues in the test-sets. The definitions of TP, FP, FN, TN, SENS, SPEC, COV, and F-measure are same as Table 5. Residues only from the first cluster of predicted residues were considered in this analysis. TP: True Positive, FP: False Positive, FN: False Negative, TN: True Negative, SENS: Sensitivity, SPEC: Specificity, COV: Coverage.

### The Effect of Discarding Residues Involved in the Protein–Protein Interactions

We found that the number of functional residues masked and discarded (%Mask) from the substitution table does not always guarantee the best performance (SENS) of ESST in detecting functional sites using CRESCENDO. The rank correlation between %Mask and SENS is 0.45 (see Table 3). Hence, it is very evident that masking-models outperform non-masking and the ESST of Shi *et al.* as described above. However the category of functional residues does matter and affects the performance. Figure 2 shows the performance of 17 ESSTs on the predictions of 602 active sites of the test-sets. Regardless of the alignment source, the performance (Z-score and SENS) of table B (no-PPI mask) is always better than table A (all mask), which means discarding PPI residues is not effective in the recognition performance of enzyme's active sites. In addition, OLD-B also outperforms OLD-J by 5% in the sensitivity, where the difference lies in the PPI residues as well. However, in the case of recognizing PPI residues, table A of ALL-matrix outperforms table B by 5.2% in terms of TP (Table 6). Interestingly, table C, which does not mask active sites, ranked as second highest and the performance of table D, which masks only active sites, is worse than the random-masking (R) substitution table (see Table 6). This result indicates that discarding PPI residues can increase the recognition performance of PPI residues but does not improve predictions of active sites of enzymes. This observation probably arises from the fact that the interfacial interactions differ in nature from those residues in catalytic sites and therefore masking of catalytic residues has little impact on those in interfaces.

### Concluding Remarks

We have shown that discarding functional residues from the calculation of the substitution table improves the detection of functional residues when the new substitution table is used with CRESCENDO. We considered four categories of functional residues in this study (Table 1) and found that functional residues can be best predicted when the relevant category is discarded from the calculation of the substitution table. Our new masking models outperformed non-masking, random masking and the old ESST (Shi *et al.*, [11]) not only in terms of true positives but also sensitivity. However, as shown in Tables 5 and 6, false positives (FPs) and false negatives (FNs) are relatively high compared with the number of true positives (TPs). The reason for high FPs is expected to arise from the restricted definition of functional residues. As shown in Figure 3A, FPs, coloured in green, are clustered around the catalytic triad (CYS-HIS-ASP) of the cysteine protease shown here. Some of these residues will be important for the local architecture of the active site and may even be buried; the substitutions accepted at these positions will therefore be restrained. Others will be directly involved in binding and positioning the substrate for catalysis. We have previously shown that CRESCENDO identifies such residues in predicting the active site [8]. Furthermore we have shown that the degree of residue conservation is significantly higher the closer the residues are to the active site and that geometrical proximity to the known active sites can be considered to constitute a new environment of ESST [12]. A reason for some high FNs is that we took only the first cluster predicted by CRESCENDO into account as positive results in the benchmark analysis; however CRESCENDO is expected to predict all regions under functional restraints and occasionally those critical for protein interactions, allostery, metal binding, post-translational modification and so on will be as conserved and score as high or higher than the active site residues. In addition, the annotations of functional residues might not be complete, which makes both FPs and FNs relatively high.

Other than CRESCENDO, there are several computational approaches to detecting possible functional regions of a protein in a fast and low-cost manner. Among them, the Evolutionary Trace method (ET), introduced by Lichtarge *et al.* [25] in 1996, is widely used and very successful in identifying functional regions, for example of SH2, SH3, and DNA binding domains. ET differs from CRESCENDO in that it identifies conserved residues only on the protein surface and exploits the use of a phylogenetic tree to identify local patterns of conservation unique but distinct amongst different branches which constitute protein subfamilies. Hence, the performance of ET highly depends on the quality of a phylogenetic tree which is determined by a set of sequences to which a query protein belongs. If the set of sequences were recently diverged, the branch-specific conservation could not be detected because the substitutions were not accumulated enough to construct a reasonable phylogenetic tree. CRESCENDO does not explicitly use the phylogenetic tree (although it could well do so), but will also not work well if the degree of divergence is low. It will, however, gain from local conservation of buried residues in the active site, for example the threonine of the aspartic proteinase catalytic triad. It also gains from a careful definition of the expected substitution patterns in any local environment and for this the proper treatment of functional residues when deriving substitution tables is of critical importance.

## Materials and Methods

### Structure Alignments

New ESSTs were derived from the structure alignments of SCOP families [19]. Baton (D.F. Burke, unpublished, Table S4), which is a successor of COMPARER [26], was used as a structure alignment program. The domain boundary and classification scheme of protein families were adopted from SCOP 1.71 as of this writing. PDB [27] was used as a source for protein three-dimensional structures. SCOP class F, which contains membrane and cell surface proteins, was not included in the alignment process as their amino acids can be in environments which differ from those in the cytoplasm. Also, non-canonical SCOP classes, H, I, J, and K, which are coiled-coil proteins, low resolution protein structures, peptides, and designed proteins, respectively, were removed from the alignment sources.

To guarantee the best alignment quality, the following three filtering conditions were applied. (1) Filtering by resolution: NMR structures and structures having resolution worse than 2.5 Å were not included in the alignment procedures. (2) Filtering by sequence identity: For each SCOP family, protein domains were clustered by running CD-HIT [28] with sequence identity of 80% or more. Within a cluster, a protein structure having the best resolution was selected as the representative. This is to remove any bias arising from the majority sequences of proteins in a SCOP family. (3) Filtering by sequence length: Within a SCOP family, the average sequence length is maintained by removing any domains having sequence below (1−0.3)*mean-length and above (1+0.3)*mean-length. Single member SCOP families were removed as they can not provide multiple alignments for the substitution calculation.

### Mapping UniProt and PDB at Residue Level

To take advantage of UniProt annotations in terms of three-dimensional structures, we developed a mapping protocol, “double-map”, which aligns a sequence of UniProt with that of PDB at residue level. Three sequences are required for every PDB chain; 1) one from SEQRES record of a PDB file, 2) another from the residue (SEQ) in ATOM record of a PDB file, and 3) the third (SP) from the corresponding UniProt entry of a PDB chain. Double-map makes two alignments from the three sequences (so the name “double-map”). The first is an alignment between SEQ and SEQRES and the second is between SEQRES and SP. Using SEQRES as a reference, SP can be aligned with SEQ and the locations of UniProt residues can be mapped onto three-dimensional structures. Ideally, the alignment between SEQ and SP is enough to locate UniProt residues in PDB. However, residues in the sequence (SEQRES) can be absent and sometimes different from the coordinate section (SEQ) for various reasons (e.g., the position in space is undetermined) and this makes the direct alignment between SEQ and SP incomplete. Double-map uses two sequence alignment programs; EXONERATE [29] and BL2SEQ of NCBI blast package [30]. If EXONERATE fails to run for a short sequence around 10–15 amino acids, BL2SEQ succeeds to complete the alignment.

### Calculation of Substitutions and Distance of Substitution Table

The program SUBST (http://www-cryst.bioc.cam.ac.uk/kenji/subst), written by Dr Kenji Mizuguchi (unpublished software, Table S4), was used in the calculation of substitution table. SUBST takes structural templates as inputs which can be generated by JOY [31], a program to identify the local structural environments of amino acids in the structure alignment files. The Euclidean distance between two ESSTs, X and Y, (DIST(X·Y)) was calculated as; 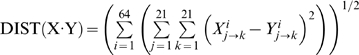, where 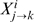 and 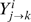 is the probability of amino acid *j* to be substituted by *k* from the ESST of X and Y under the structure environment of *i*. Note that there are 64 structure environments (4*2*8 from the secondary structures, solvent accessibility and H-bonds, respectively) and 21 amino acids (Cysteine and half-cysteine using one-letter code J and C, respectively).

### Benchmarking

CRESCENDO [8] was used to benchmark new ESSTs based on the predictions of four categories of functional residues: (1) catalytic residues of enzyme active sites, (2) residues involved in protein–protein interactions, (3) protein–nucleic acid interactions, and (4) protein–ligand interactions (see Table 1 for the source). The divergent score was used as it is more sensitive to the environments and it better discriminates functionally conserved residues from structurally conserved residues. The CRESCENDO scores (Z-score) were smoothed and contoured using Kin3Dcont [32]. CRESCENDO returns several clusters of predicted residues based on the size of grid points contoured using the Z-score. Residues only in the first cluster were used as the predicted residues of functional residues in the analysis. The details of the equation can be found in the original paper [8]. The P-value of the predicted residues is calculated using a one-tailed test under the standard normal distribution.

The performance ESSTs were assessed by measuring sensitivity (SENS), coverage (COV) and F-measure. These measurements were calculated based on the ratios derived from TP (true positives), FP (false positives), FN (false negatives), and TN (true negatives), which are defined as follow.
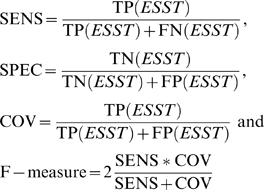



TP is the number of residues correctly predicted by CRESCENDO. If the residues predicted by CRESCENDO are the same as those annotated by the reference database, they are counted as being correct. FN is the number of real functional residues where CRESCENDO failed to predicted. FP is the number of false hits that CRESCENDO predicted as functional residues but not actually annotated by the references. TP, FP, FN, and TN are exclusively determined by the ESST used in CRESCENDO.

The Spearman's rank correlation (*ρ*) was calculated as follows; 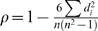, where *d_i_* is the difference between each rank of corresponding values and *n* is the number of pairs of values.

## Supporting Information

Table S1 Probability of Residue Conservation.
(0.11 MB DOC)

Table S2 Distance Matrix of 17 ESSTs.
(0.10 MB DOC)

Table S3 Performance of ESSTs on the Residue Interacting with Nucleic Acids and Ligands.
(0.07 MB DOC)

Table S4 Lists of Computer Programs and Databases used in this Study.
(0.06 MB DOC)
