# Supplementary material for: Discarding Functional Residues from the Substitution Table Improves Predictions of Active Sites within Three-Dimensional Structures
Source: PLoS Comput Biol. 2008 Oct 3;4(10):e1000179. doi: 10.1371/journal.pcbi.1000179 (PMC2527532; doi:10.1371/journal.pcbi.1000179)
Supplement: Table S1 — Probability of Residue Conservation. (0.11 MB DOC) [file pcbi.1000179.s001.doc]

**Table S1. Probability of Residue Conservation**

For each masking type, the diagonal entries (not substituted entries) are averaged over 64 ESSTs. Note that there are 21 amino acids (J for cysteine and C for half-cysteine) in this table. P_CONS_ in Table 3 is the average probability (AVG) in the bottom line.

(^1^: 21 amino acids, ^2^: Masking-types, ^3^: Matrix-types, ^4^: average)

|  | **OLD^3^** |  |  |  | **ENZ** |  |  |  |  |  | **NOENZ** | **ALL** |  |  |  |  |  |
| --- | --- | --- | --- | --- | --- | --- | --- | --- | --- | --- | --- | --- | --- | --- | --- | --- | --- |
|  | **X^2^** | **J** | **B** | **R** | **X** | **A** | **B** | **C** | **D** | **R** | **X** | **X** | **A** | **B** | **C** | **D** | **R** |
| **A^1^** | 29.84 | 30.08 | 29.84 | 29.72 | 25.84 | 26.92 | 26.02 | 26.89 | 25.88 | 25.98 | 23.66 | 23.36 | 23.90 | 23.25 | 23.89 | 23.38 | 23.33 |
| **C** | 76.94 | 77.00 | 63.29 | 77.28 | 60.51 | 60.77 | 59.41 | 61.32 | 59.77 | 60.11 | 84.57 | 75.90 | 75.95 | 75.98 | 76.04 | 75.81 | 74.33 |
| **D** | 44.89 | 44.52 | 41.34 | 45.07 | 41.38 | 38.16 | 37.91 | 40.57 | 38.91 | 42.31 | 35.84 | 38.39 | 36.01 | 35.32 | 37.03 | 37.39 | 38.65 |
| **E** | 32.36 | 32.17 | 29.16 | 32.15 | 33.27 | 31.95 | 31.27 | 33.72 | 31.22 | 33.36 | 27.83 | 29.66 | 28.72 | 27.92 | 29.66 | 28.51 | 30.10 |
| **F** | 33.99 | 32.71 | 34.14 | 33.88 | 26.07 | 25.60 | 25.26 | 25.68 | 26.09 | 26.19 | 25.00 | 23.53 | 23.96 | 23.32 | 24.07 | 23.54 | 23.97 |
| **G** | 53.50 | 53.00 | 52.32 | 53.81 | 53.24 | 50.74 | 49.63 | 50.77 | 53.25 | 53.73 | 45.99 | 47.32 | 45.83 | 45.19 | 45.84 | 47.32 | 47.30 |
| **H** | 38.72 | 32.06 | 28.39 | 38.90 | 33.31 | 32.36 | 31.20 | 33.88 | 31.20 | 34.29 | 24.83 | 24.78 | 23.30 | 22.66 | 24.14 | 23.85 | 25.60 |
| **I** | 26.61 | 26.94 | 26.54 | 26.32 | 23.25 | 23.38 | 22.94 | 23.36 | 23.28 | 23.85 | 21.10 | 20.94 | 21.05 | 20.67 | 21.06 | 20.94 | 20.85 |
| **J** | 31.33 | 15.68 | 17.45 | 32.03 | 15.20 | 11.86 | 11.30 | 15.10 | 11.84 | 14.95 | 16.79 | 14.72 | 9.31 | 9.37 | 11.32 | 13.36 | 14.30 |
| **K** | 34.03 | 34.05 | 28.59 | 33.78 | 38.20 | 33.40 | 32.96 | 33.22 | 38.19 | 37.54 | 24.00 | 33.01 | 27.33 | 27.08 | 27.68 | 32.68 | 32.34 |
| **L** | 36.04 | 35.89 | 36.10 | 36.08 | 31.36 | 31.99 | 31.10 | 31.95 | 31.39 | 31.81 | 30.41 | 29.25 | 29.86 | 29.16 | 29.87 | 29.27 | 29.54 |
| **M** | 18.13 | 17.86 | 17.27 | 17.96 | 15.70 | 16.17 | 15.85 | 16.15 | 15.73 | 15.28 | 10.61 | 11.50 | 11.19 | 11.32 | 11.22 | 11.51 | 11.73 |
| **N** | 30.16 | 29.96 | 28.19 | 30.33 | 30.97 | 31.56 | 30.65 | 31.74 | 30.79 | 29.92 | 22.36 | 25.93 | 25.69 | 25.13 | 25.77 | 25.88 | 26.02 |
| **P** | 45.46 | 45.55 | 45.45 | 45.48 | 43.80 | 44.29 | 43.88 | 44.30 | 43.82 | 44.35 | 39.01 | 38.43 | 38.62 | 38.48 | 38.58 | 38.43 | 38.61 |
| **Q** | 24.89 | 24.85 | 24.99 | 25.04 | 20.29 | 20.07 | 20.42 | 20.11 | 20.28 | 20.40 | 16.90 | 16.63 | 16.20 | 16.51 | 16.25 | 16.63 | 16.62 |
| **R** | 35.70 | 34.70 | 33.74 | 35.54 | 40.00 | 40.42 | 40.05 | 40.51 | 39.87 | 40.46 | 22.97 | 30.45 | 29.82 | 30.17 | 29.96 | 30.36 | 31.42 |
| **S** | 29.10 | 29.00 | 28.07 | 29.21 | 25.36 | 23.62 | 23.54 | 24.35 | 24.65 | 25.17 | 18.34 | 21.74 | 21.00 | 20.67 | 21.29 | 21.49 | 21.98 |
| **T** | 30.08 | 29.95 | 28.94 | 30.00 | 24.88 | 23.21 | 22.78 | 23.31 | 24.87 | 24.60 | 21.79 | 22.62 | 22.38 | 21.84 | 22.40 | 22.61 | 22.87 |
| **V** | 30.05 | 29.97 | 30.05 | 30.11 | 27.56 | 26.29 | 26.00 | 26.27 | 27.58 | 27.52 | 24.69 | 24.12 | 24.24 | 23.75 | 24.23 | 24.13 | 24.16 |
| **W** | 49.62 | 49.68 | 50.04 | 49.43 | 34.52 | 36.31 | 34.39 | 36.27 | 34.56 | 36.34 | 43.62 | 32.81 | 34.15 | 32.83 | 34.16 | 32.84 | 33.37 |
| **Y** | 39.95 | 39.63 | 39.55 | 40.15 | 29.07 | 29.07 | 28.50 | 30.07 | 28.23 | 29.52 | 27.11 | 25.92 | 25.18 | 25.68 | 25.64 | 25.59 | 25.71 |
| **AVG^4^** | 36.73 | 35.49 | 33.97 | 36.77 | 32.08 | 31.34 | 30.72 | 31.88 | 31.49 | 32.27 | 28.93 | 29.10 | 28.27 | 27.92 | 28.58 | 28.83 | 29.18 |
