# Supplementary material for: Discarding Functional Residues from the Substitution Table Improves Predictions of Active Sites within Three-Dimensional Structures
Source: PLoS Comput Biol. 2008 Oct 3;4(10):e1000179. doi: 10.1371/journal.pcbi.1000179 (PMC2527532; doi:10.1371/journal.pcbi.1000179)
Supplement: Table S2 — Distance Matrix of 17 ESSTs. (0.10 MB DOC) [file pcbi.1000179.s002.doc]

**Table S2. Distance Matrix of 17 ESSTs**

The difference between ESSTs is measured by the distance defined in Materials and Methods. DIST in Table 5 is same as the first row of this table.

Within a pair of matrix-type (OLD, ENZ, NOENZ and ALL), two farthest distances are in a bold character. Pairs of matrix-type are shaded alternately.

| **Matrix Type^1^** |  | **OLD** |  |  |  | **ENZ** |  |  |  |  |  | **NOENZ** | **ALL** |  |  |  |  |  |
| --- | --- | --- | --- | --- | --- | --- | --- | --- | --- | --- | --- | --- | --- | --- | --- | --- | --- | --- |
|  | **Masking Type ^2^** | **X** | **J** | **B** | **R** | **X** | **A** | **B** | **C** | **D** | **R** | **X** | **X** | **A** | **B** | **C** | **D** | **R** |
| **OLD** | **X** | 0.0 | 170.7 | **220.6** | 33.8 | 464.0 | 481.6 | **487.0** | 465.6 | 481.6 | 460.6 | **464.9** | 466.1 | 489.2 | **496.3** | 476.9 | 474.7 | 459.5 |
|  | **J** |  | 0.0 | 161.3 | 177.9 | 437.5 | 443.0 | 446.8 | 437.1 | 444.2 | 433.6 | 428.6 | 428.2 | 433.6 | 441.7 | 426.8 | 432.7 | 420.6 |
|  | **B** |  |  | 0.0 | **226.1** | 430.5 | 427.4 | 426.1 | 425.4 | 432.0 | 425.7 | 435.5 | 435.5 | 432.5 | 438.6 | 427.8 | 437.5 | 424.5 |
|  | **R** |  |  |  | 0.0 | 465.3 | 482.7 | **488.4** | 466.4 | 483.2 | 461.8 | **465.5** | 467.7 | 491.1 | **498.2** | 478.7 | 476.5 | 461.1 |
| **ENZ** | **X** |  |  |  |  | 0.0 | 145.8 | 133.0 | 124.8 | 74.9 | 84.3 | **507.0** | 340.4 | 356.1 | 365.5 | 345.0 | 348.2 | 322.6 |
|  | **A** |  |  |  |  |  | 0.0 | 74.9 | 73.3 | 124.3 | **147.6** | 501.2 | 363.5 | 340.3 | 356.5 | 338.0 | 364.1 | 342.5 |
|  | **B** |  |  |  |  |  |  | 0.0 | 107.2 | 104.7 | **146.8** | 492.4 | 344.3 | 325.8 | 334.8 | 324.2 | 344.1 | 324.5 |
|  | **C** |  |  |  |  |  |  |  | 0.0 | 141.6 | 128.3 | 502.2 | 361.6 | 351.1 | 366.7 | 340.7 | 368.1 | 341.1 |
|  | **D** |  |  |  |  |  |  |  |  | 0.0 | 109.6 | 505.3 | 342.2 | 343.8 | 353.4 | 340.6 | 343.0 | 323.7 |
|  | **R** |  |  |  |  |  |  |  |  |  | 0.0 | **508.3** | 357.3 | **367.8** | **378.9** | 357.5 | 364.4 | 337.5 |
| **NOENZ** | **X** |  |  |  |  |  |  |  |  |  |  | 0.0 | **310.8** | 309.0 | 303.1 | 306.9 | 308.8 | **315.9** |
| **ALL** | **X** |  |  |  |  |  |  |  |  |  |  |  | 0.0 | **147.0** | 130.7 | 132.7 | 37.9 | 73.1 |
|  | **A** |  |  |  |  |  |  |  |  |  |  |  |  | 0.0 | 70.8 | 44.3 | 136.2 | **147.5** |
|  | **B** |  |  |  |  |  |  |  |  |  |  |  |  |  | 0.0 | 83.7 | 117.5 | 141.4 |
|  | **C** |  |  |  |  |  |  |  |  |  |  |  |  |  |  | 0.0 | 132.9 | 134.6 |
|  | **D** |  |  |  |  |  |  |  |  |  |  |  |  |  |  |  | 0.0 | 81.1 |
|  | **R** |  |  |  |  |  |  |  |  |  |  |  |  |  |  |  |  | 0.0 |

(^1^: Matrix type in Table 2, ^2^: Masking type in Table 2)
