# Supplementary material for: Discarding Functional Residues from the Substitution Table Improves Predictions of Active Sites within Three-Dimensional Structures
Source: PLoS Comput Biol. 2008 Oct 3;4(10):e1000179. doi: 10.1371/journal.pcbi.1000179 (PMC2527532; doi:10.1371/journal.pcbi.1000179)
Supplement: Table S3 — Performance of ESSTs on the Residue Interacting with Nucleic Acids and Ligands. (0.07 MB DOC) [file pcbi.1000179.s003.doc]

**Table S3. Performance of ESSTs on the Residue Interacting with Nucleic-acids and Ligands**

Out of 81,410 residues in the test-sets, 194 residues are annotated as DNA_BIND by UniProt [1] or BIPA and 1348 residues are annotated as either BINDING, CA_BIND, NP_BIND or METAL by UniProt (see Table 1 for the annotations). For those residues, if CRESCENDO [2] could correctly predict, they were counted as TP.

| **Matrix Type** | **Masking Type** | **PNI^a^** |  | **PLI^b^** |  |
| --- | --- | --- | --- | --- | --- |
|  |  | **TP** | **SENS** | **TP** | **SENS** |
| **OLD** | B | 20 | 0.1031 | 274 | 0.2033 |
|  | J | 25 | 0.1289 | 261 | 0.1936 |
|  | R | 22 | 0.1134 | 261 | 0.1936 |
|  | X | 22 | 0.1134 | 253 | 0.1877 |
| **ENZ** | At | 24 | 0.1237 | 259 | 0.1921 |
|  | Bt | 25 | 0.1289 | 265 | 0.1966 |
|  | Ct | 25 | 0.1289 | 254 | 0.1884 |
|  | Dt | 27 | 0.1392 | 261 | 0.1936 |
|  | Rt | 22 | 0.1134 | 260 | 0.1929 |
|  | Xt | 22 | 0.1134 | 259 | 0.1921 |
| **NOENZ** | X | 32 | 0.1649 | 279 | 0.2070 |
| **ALL** | At | 27 | 0.1392 | 281 | 0.2085 |
|  | Bt | 34 | 0.1753 | 283 | 0.2099 |
|  | Ct | 27 | 0.1392 | 286 | 0.2122 |
|  | Dt | 40 | 0.2062 | 280 | 0.2077 |
|  | Rt | 34 | 0.1753 | 277 | 0.2055 |
|  | Xt | 35 | 0.1804 | 291 | 0.2159 |

(^a^: Protein-nucleic acid interaction sites, ^b^: Protein-ligand interaction sites, TP: True Positive, SENS: Sensitivity)
