# Supplementary material for: Discarding Functional Residues from the Substitution Table Improves Predictions of Active Sites within Three-Dimensional Structures
Source: PLoS Comput Biol. 2008 Oct 3;4(10):e1000179. doi: 10.1371/journal.pcbi.1000179 (PMC2527532; doi:10.1371/journal.pcbi.1000179)
Supplement: Table S4 — Lists of Computer Programs and Databases used in this Study. (0.06 MB DOC) [file pcbi.1000179.s004.doc]

**Table S4. Lists of Computer Programs and Databases used in this Study**

This table is also available from http://www-cryst.bioc.cam.ac.uk/ESST.

| **Category** | **Name** | **Description** | **URL** |
| --- | --- | --- | --- |
| **Software** | BATON | Structure alignments | http://www-cryst.bioc.cam.ac.uk/COMPARER |
|  | CRESCENDO | Detecting functionally important residues | http://www.bioinf.manchester.ac.uk/crescendo |
|  | SUBST | ESST calculation | http://www-cryst.bioc.cam.ac.uk/~kenji/subst |
|  | JOY | Protein structure and alignment analysis | http://www-cryst.bioc.cam.ac.uk/~joy |
|  | Kin3DCont | Making contour maps in kinemage format | http://kinemage.biochem.duke.edu/software/kincon.php |
|  | EXONERATE | A generic tool for sequence alignment | http://www.ebi.ac.uk/~guy/exonerate/ |
|  | BL2SEQ | This tool produces the alignment of two given sequences | http://www.ncbi.nlm.nih.gov/blast/bl2seq/wblast2.cgi |
|  | CD-HIT | A program for clustering large protein database at high sequence identity threshold | http://bioinformatics.ljcrf.edu/cd-hi/ |
| **Database** | CSA | Catalytic Site Atlas | http://www.ebi.ac.uk/thornton-srv/databases/CSA |
|  | HOMSTRAD | Homologous Structure Alignment Database | http://tardis.nibio.go.jp/homstrad |
|  | InterPare | A database server for protein interaction interfaces | http://interpare.net |
|  | SCOP | Structural Classification of Proteins | http://scop.mrc-lmb.cam.ac.uk/scop |
|  | UniProt | A comprehensive protein sequences and annotations | http://uniprot.org |
